# Genome-wide identification and expression analysis of the *ftsH* protein family and its response to abiotic stress in *Nicotiana tabacum* L

**DOI:** 10.1186/s12864-022-08719-x

**Published:** 2022-07-12

**Authors:** Tianxiunan Pu, Zejun Mo, Long Su, Jing Yang, Ke Wan, Linqi Wang, Renxiang Liu, Yang Liu

**Affiliations:** Guizhou Province, College of Tobacco Science of Guizhou University/ Guizhou Key Laboratory for Tobacco Quality, Huaxi District, Guiyang City, 550025 People’s Republic of China

**Keywords:** *FtsH* family, Gene expression patterns, Abiotic stress

## Abstract

**Background:**

The filamentous temperature-sensitive H protease (*ftsH*) gene family plays an important role in plant growth and development. *FtsH* proteins belong to the AAA protease family. Studies have shown that it is a key gene for plant chloroplast development and photosynthesis regulation. In addition, the *ftsH* gene is also involved in plant response to stress. At present, the research and analysis of the *ftsH* gene family are conducted in microorganisms such as *Escherichia coli* and Oenococcus and various plants such as Arabidopsis, pear, rice, and corn. However, analysis reports on *ftsH* genes from tobacco (*Nicotiana tabacum* L.), an important model plant, are still lacking. Since *ftsH* genes regulate plant growth and development, it has become necessary to systematically study this gene in an economically important plant like tobacco.

**Results:**

This is the first study to analyze the *ftsH* gene from *Nicotiana tabacum* L. K326 (*NtftsH*). We identified 20 *ftsH* genes from the whole genome sequence, renamed them according to their chromosomal locations, and divided them into eight subfamilies. These 20 *NtftsH* genes were unevenly distributed across the 24 chromosomes. We found four pairs of fragment duplications. We further investigated the collinearity between these genes and related genes in five other species. Quantitative real-time polymerase chain reaction (qRT-PCR) analysis identified differential expression patterns of *NtftsH* in different tissues and under various abiotic stress conditions.

**Conclusions:**

This study provides a comprehensive analysis of the *NtftsH* gene family. The exon–intron structure and motif composition are highly similar in *NtftsH* genes that belong to the same evolutionary tree branch. Homology analysis and phylogenetic comparison of *ftsH* genes from several different plants provide valuable clues for studying the evolutionary characteristics of *NtftsH* genes. The *NtftsH* genes play important roles in plant growth and development, revealed by their expression levels in different tissues as well as under different stress conditions. Gene expression and phylogenetic analyses will provide the basis for the functional analysis of *NtftsH* genes. These results provide a valuable resource for a better understanding of the biological role of the *ftsH* genes in the tobacco plant.

**Supplementary Information:**

The online version contains supplementary material available at 10.1186/s12864-022-08719-x.

## Background

Chloroplasts are the site of photosynthesis in plants. The processing, maturation, and degradation of proteins in chloroplasts are important for normal plant growth and development and a common regulatory mechanism towards environmental changes [1]. Chloroplast proteases play an important role in this process, which maintains the stability of the protein system in the chloroplast and ensures the orderly progress of the normal life activities of plants. The proteases in plant chloroplasts are divided into three categories: metalloproteases, serine proteases, and aspartic proteases [2]. *FtsH*, a type of metalloprotease originally discovered in *Escherichia coli*, belongs to the AAA family of proteins. It is an ATP-dependent metalloproteinase encoded by the *ftsH* gene, with ATPase, proteolysis, and chaperone activity [3, 4]. The members of the *FtsH* family aggregate to form functional homohexamers or heterohexamers. Each *ftsH* protease contains an N-terminal transmembrane segment and a C-terminal region composed of the AAA-ATPase domain that belongs to the M41 peptidase family of protease domains [5, 6].

The FtsH protease plays an important role in eukaryotes and is generally found in mitochondria and chloroplasts. Several studies have shown that *ftsH* protease participates in the initial cleavage of D1-binding protein in the reaction center of photosystem II, thereby reducing the damage to the photosynthetic system from high-intensity light [7–10]. In addition, under the combined stress condition of high temperature and strong light, Deg1 and *ftsH* proteases participate in the regulation of D1 protein turnover. Recent studies have found that *ftsH* protease is essential for chloroplast development. An *ftsH* rice mutant plant show ed albino traits [11, 12]. In addition, *ftsH* protease plays an important role during stress conditions such as drought, high temperature, long exposure to ultraviolet light, and high salt concentration [10, 13–16].

As a model organism for studying the basic biological processes of plants, tobacco is also a source of plant BY-2 cell lines and an important tool for plant molecular research. Like other nightshade plants, including potatoes, tomatoes, and peppers, it is also used as a model for plant disease susceptibility, and tobacco is an important commercial crop. The growth and development of tobacco are affected by the environment showing varied growth rates under different environmental conditions. An unsuitable environment can inhibit tobacco growth and even lead to plant death.

At present, the analytical research on the *ftsH* gene family is carried out on a genome-wide scale that involves many organisms, including microorganisms such as *Lactobacillus plantarum*, *Escherichia coli*, *Bacillus subtilis*, cyanobacteria, *Acidovorax citrulli* [4, 9, 17–20], and plants such as Arabidopsis, pear, rice, corn, and peanut [14, 21–25]. However, no report on the ftsH gene family has been found in tobacco. Because of the importance of the ftsH gene family in the process of plant growth and development, it is also to screen out new resistance genes and provide a basis for the molecular mechanism of tobacco resistance to stress. Therefore, it is important to systematically study the tobacco ftsH gene family. This study performed a comprehensive analysis of the *NtftsH* gene family, including gene structure, motif composition, chromosomal location, and phylogenetic tree. The evolutionary relationship of tobacco with multiple species was established by sequence comparison, and the expression profile of *FtsH* under different stress conditions was analyzed. Therefore, this study puts across an in-depth understanding of the function of the *ftsH* gene family, specifically its role in stress resistance in tobacco.

## Results

### Identification of tobacco *ftsH* gene

From tobacco's whole genome sequence data, we identified 20 members of the *ftsH* gene family (Additional file 1: Table S1). The genes were renamed according to their order and position on the chromosome, from NtftsH1 to NtftsH20 [26]. At the same time, the physicochemical properties and subcellular localization of gene sequences were analyzed. *NtftsH*9 is the smallest protein with 456 amino acids (aa), while *NtftsH*3 is the largest protein with 1239 amino acids (aa); the molecular weights of proteins range from 51.15 (*NtftsH*9) to 135.53 kDa (*NtftsH*3), the Pi ranged from 5.61 (*NtftsH*13) to 11.17 (*NtftsH*18).

The results of subcellular localization showed that except few (*NtftsH*3, *NtftsH*7, *NtftsH*13, and *NtftsH*17), the *ftsH* genes are expressed in the chloroplast, many of which were specific to the chloroplast (*NtftsH*5, *NtftsH*10, *NtftsH*14, and *NtftsH*16). In addition, except for the three genes *NtftsH*5, *NtftsH*10, and *NtftsH*14, 17 other *ftsH* gene products were localized in the nucleus, out of which, three were exclusively localized in the nucleus (*NtftsH*3, *NtftsH*7, and *NtftsH*13). Finally, *NtftsH*17 is exclusively expressed in the mitochondria.

### Multiple sequence alignment and phylogenetic analysis of *NtftsH* gene

**Fig. 1:**
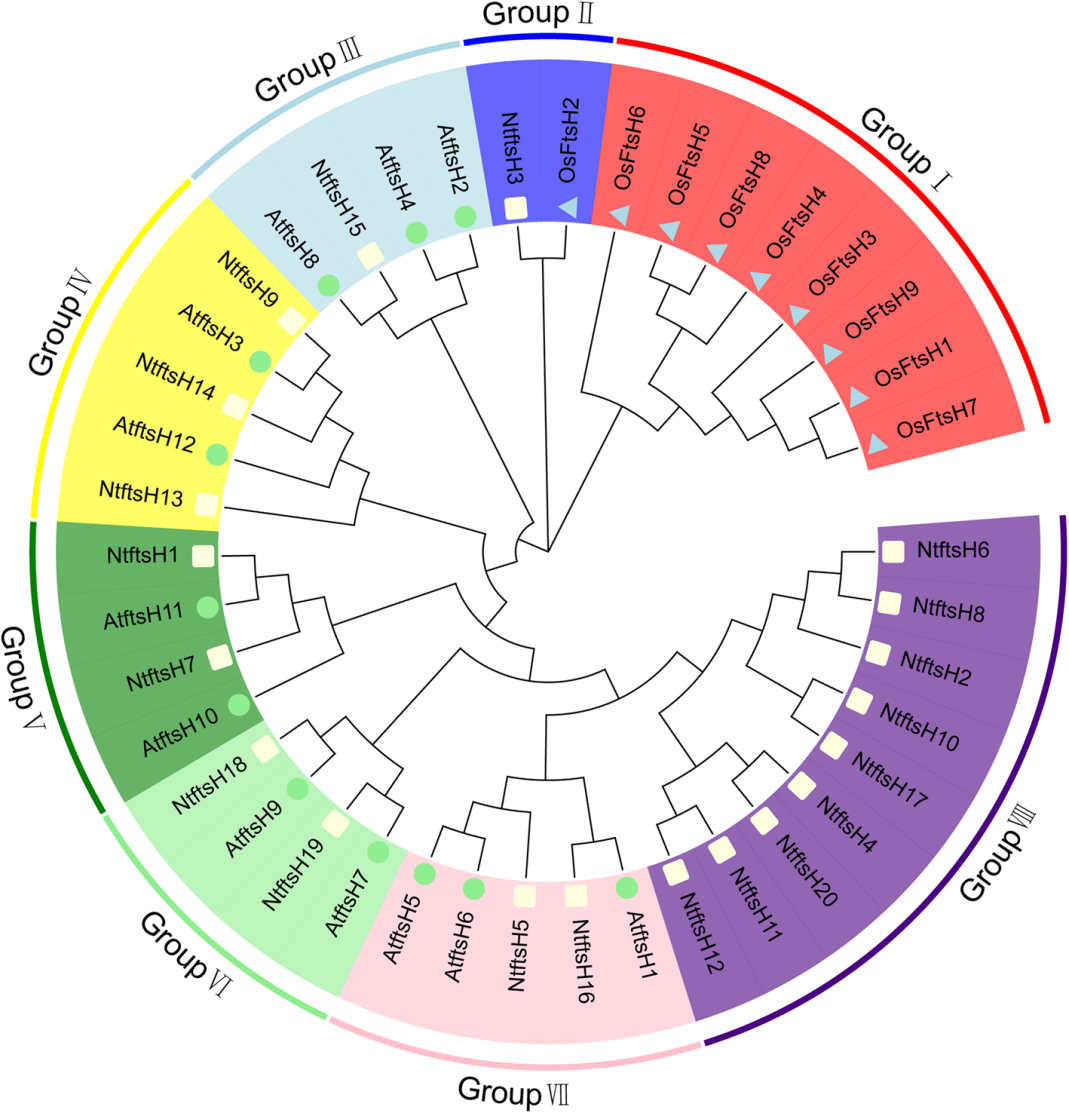
The rootless phylogenetic tree was constructed based on the gene sequences of *ftsH* from Arabidopsis, tobacco, and rice; each arc represents a group (a total of 8 groups). The circles represent the *AtftsH* gene, the rectangles represent the *NtftsH* gene, and the triangles represent the *ftsH* gene from the rice plant. *ftsH*: filamentous temperature-sensitive H

The multiple sequence alignment of tobacco *ftsH* protein sequences from tobacco (*NtftsH*) and *Arabidopsis thaliana* (*AtftsH*) showed the highly conserved regions of these proteins had specific amino-acid sequences, PGTGKT and RPGR (Additional file 2: Figure S1). *FtsH* proteins sequences from Arabidopsis (dicotyledonous), rice (monocotyledonous), and tobacco were aligned to construct a phylogenetic tree to understand the evolutionary relationship. In addition, the 41 *ftsH* genes were divided into 8 groups according to the percentage of homology between these genes. The results showed that 8 genes from rice and 9 *NtftsH* genes belonged to group I and group VIII, respectively; others were distributed from group II to group VII (Fig. 1). Compared with rice, the homology between tobacco and Arabidopsis ftsH genes is higher. This phenomenon may be due to the difference in leaf structure between tobacco, Arabidopsis and rice, resulting in differences in homology. The subcellular localization results revealed a higher homology between genes expressed in the same site either in tobacco or in Arabidopsis, which may be due to their similar function. It is logical to conclude that the *NtftsH* and *AtftsH* proteins may perform the same biological function in the cell.

### Structure and motif composition of *NtftsH* gene family

**Fig. 2:**
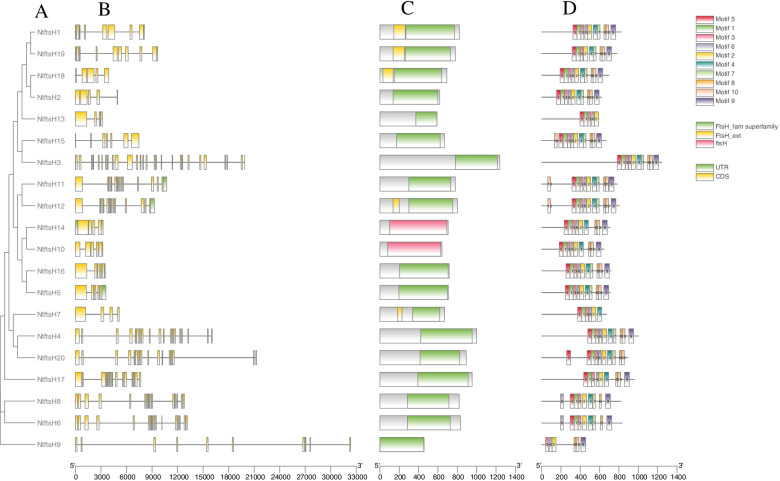
Phylogenetic relationships, gene structure analysis, and motif distributions of *Nicotiana tabacum* L. *ftsH*. *ftsH*: filamentous temperature-sensitive H. **A** Phylogenetic tree constructed by the neighbour-joining method, with 1000 replicates on each node. **B** Exons and introns are indicated by yellow rectangles, and black lines, respectively. **C** Conserved domain of *ftsH* gene. **D** Amino acid motifs in the *ftsH* proteins (1–10) are represented by coloured boxes. Black lines indicate relative protein lengths

Based on the gene annotation (GFF format), the gene structure of the *NtftsH* gene was analyzed. The results showed that all *NtftsH* genes contained multiple exons and introns (Fig. 2A,B). Also, the number and lengths of introns/exons of the genes grouped into the same branch of the evolutionary tree were roughly similar. Of these, 9 *NtftsH* genes had untranslated regions (UTRs).

All 20 *NtftsH* genes contained *FtsH*-related conserved domains — a hallmark feature of the *ftsH* protein family (Fig. 2A, C). In addition, the *ftsH* gene is not only highly conserved in chloroplasts and mitochondria but also homologous to the membrane-bound ATP-dependent protease in prokaryotes.

Using MEME online software, the composition of *NtftsH* conservative motif and the number of conservative motifs were analyzed, and a total of 10 conservative motifs were identified, which were named motif1-motif10 in turn (Fig. 2D). All tobacco *ftsH* genes contain conservative motifs 2 and 3. In addition to *NtftsH*19, the remaining 19 members of the family have developed conserved motifs6, suggesting that these motifs can serve as markers for identifying the *NtftsH* gene. At the same time, we found that genes in the same branch contain basically the same number and type of motif, and in general, the sequences of closely related members are similar, and the motif is similar. The existence of the same type of conserved motif may indicate similar functions among members of the *NtftsH* gene family, and also confirm the correct construction of the evolutionary tree.

### Chromosomal location of *NtftsH* gene and gene segment duplication events

Chromosomal location analysis was performed to further understand the distribution of *NtftsH* genes in chromosomes (Additional file 3 Figure S2). Out of 24 chromosomes (Nt1-Nt24), Nt1, Nt3, Nt4, Nt7, Nt9, Nt10, Nt11, Nt13, Nt15, Nt20, and Nt21 did not contain the *ftsH* gene; the 20 *ftsH* genes are unevenly distributed on the remaining chromosomes. Three *NtftsH* genes were mapped to chromosomes Nt17 and Nt24 each, and two *NtftsH* genes were located on each of the chromosomes Nt12, Nt16, and Nt23. The reason for this phenomenon may be due to the *ftsH* gene duplication or gene loss during the evolution of the tobacco plant, resulting in the absence of the *ftsH* gene on some chromosomes and the appearance of multiple genes on others. In addition, the frequency of gene occurrence was not related to chromosome length and gene density [26].

**Fig. 3:**
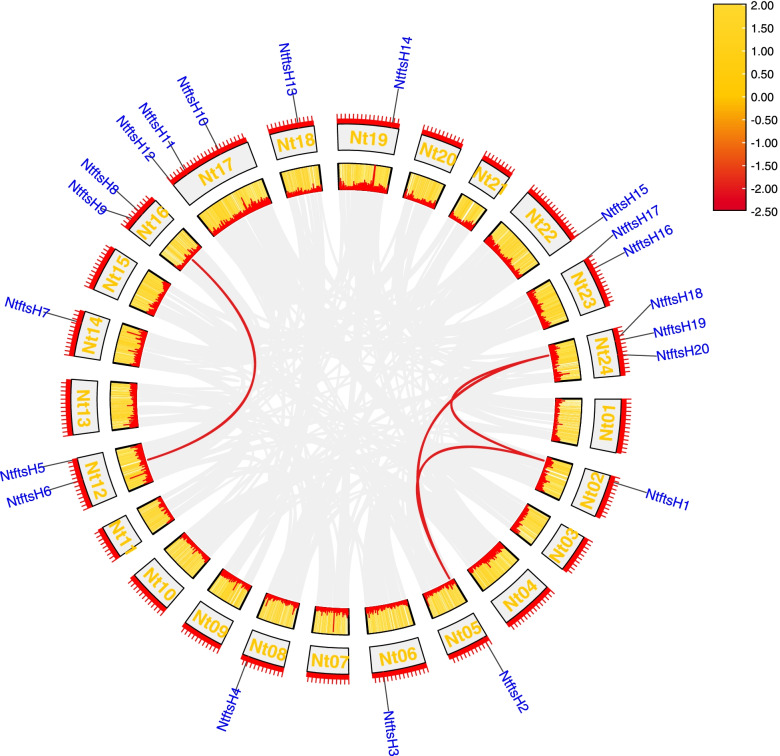
Schematic diagram of the chromosomal distribution and segmental duplication of the *NtftsH* genes. Grey lines represent all colinear blocks in the tobacco genome, and red lines represent duplicated *ftsH* gene pairs

Some studies have shown that the expansion of plant gene families is mainly facilitated by segmental and tandem duplication events [27, 28]. Segmental duplication frequently occurs during polyploidization and chromosomal rearrangements, resulting in the presence of repeated chromosomal blocks in the genome; tandem duplications are clusters of multiple adjacent homologous gene family members (two or more members) on a chromosome [29]. We explored and screened gene pairs showing segmental and tandem duplication throughout the tobacco genome to better understand gene duplication events in tobacco. According to the definition of segmental and tandem duplications, the points on the diagonal line correspond to tandem duplication gene pairs, and the rest of the points indicate segment duplication gene pairs. Our findings support that tobacco has a highly repetitive genome that contains gene pairs with a large number of segmental duplications (Fig. 3; Supplementary file 4 Table S2). In the Circos plot (Fig. 3), the four gene pairs with segmental duplications are connected by red curves. Taken together, we suggest that the expansion of the *NtftsH* gene family is related to tandem and segment duplication events, the latter being the main source of adding new members to the *NtftsH* gene family.

### Analyzation of the *cis*-acting element of the *NtftsH* gene

**Fig. 4:**
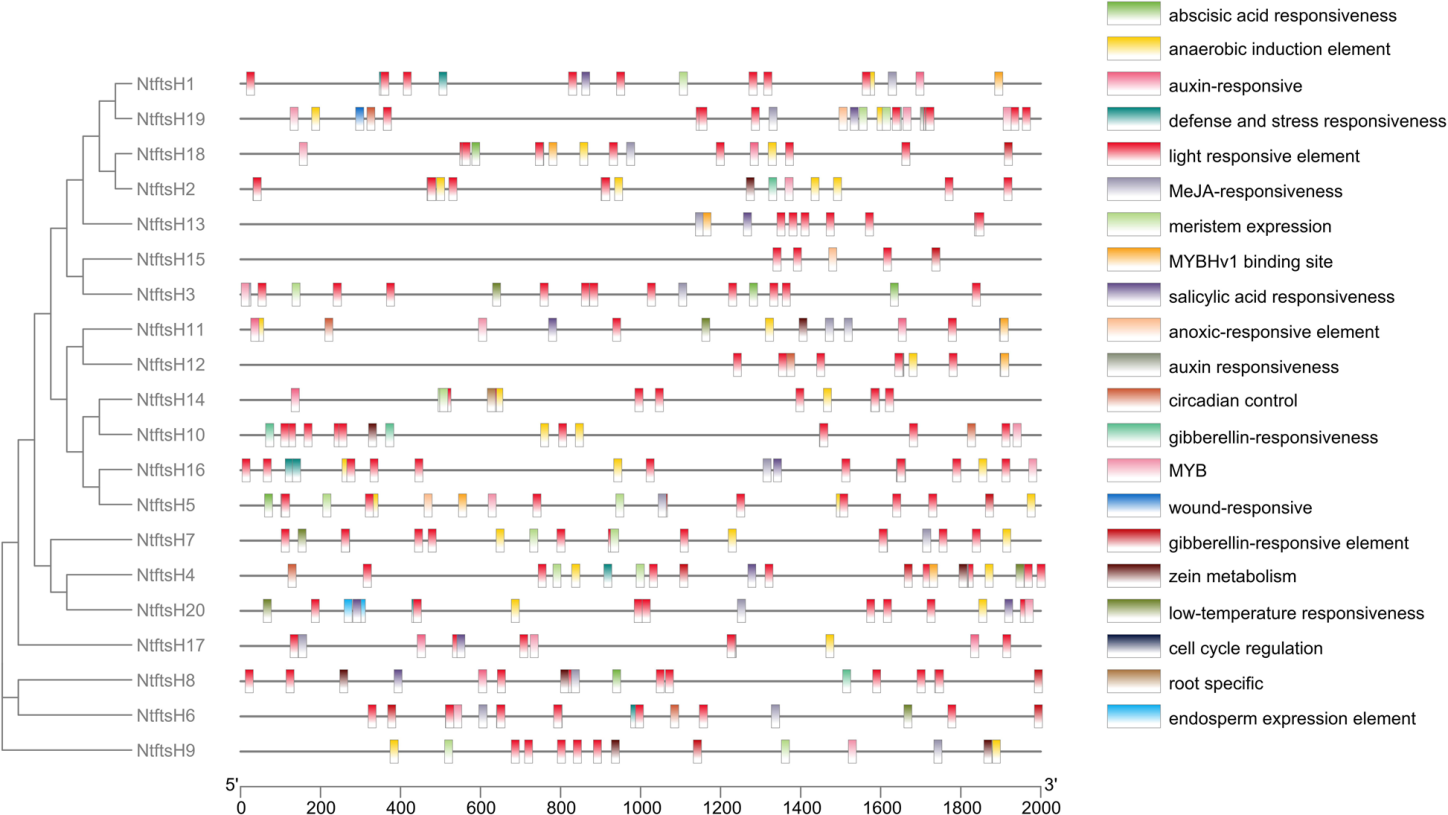
Analysis of the 2000 bp upstream *cis*-acting element of *NtftsH* gene, Diferent colors represent diferent types of *cis*-acting elements

Using the 2000 bp upstream sequence from the *NtftsH* gene promoter, *cis*-acting elements were analyzed for 20 *NtftsH* genes. According to the results (Fig. 4; Additional file 5 Table S3), *cis*-acting elements are regulated by hormones and abiotic stress conditions (drought, cold, and light). Light-responsive *cis*-acting elements were the most abundant, followed by hormone-related response elements (RE), such as abscisic acid-RE, gibberellin-RE, auxin-RE, methyl jasmonate-RE, and salicylic acid-RE. The results showed that these *cis*-acting elements also contain regions associated with the cell cycle, endosperm development, and circadian rhythm. Nociceptive RE (a *cis*-acting element that responds to biotic stress) was also identified in the tobacco *ftsH* gene promoter. It indicates that the *NtftsH* genes are closely related to plant resistance to stress conditions, including drought, low temperature, and intense light.

### Synteny analysis of *FtsH* genes

**Fig. 5:**
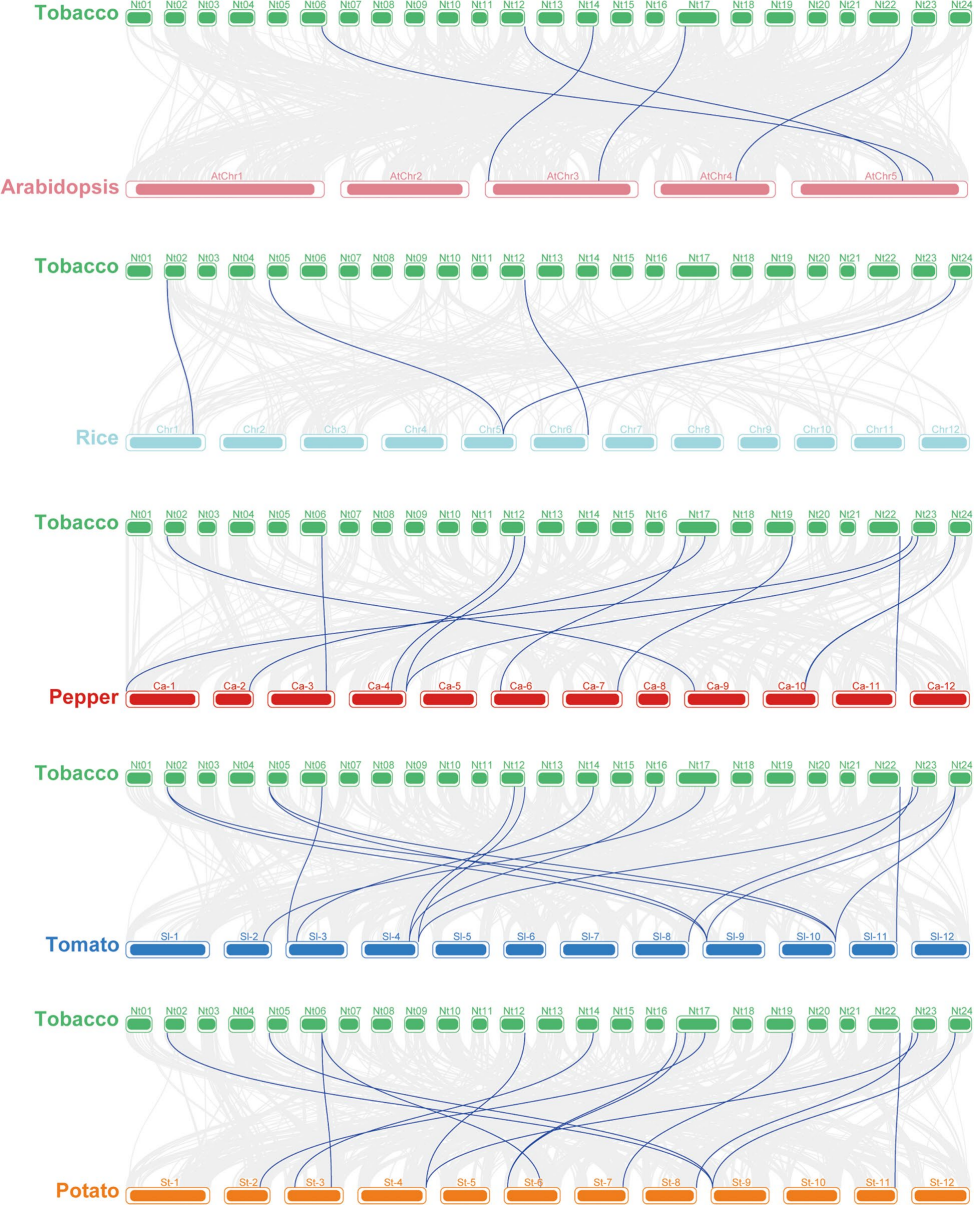
Syntenic analysis of *ftsH* genes between tobacco and five representative plant species. Gray lines in the background indicate the collinear blocks within the tobacco and other plant genomes, while the blue lines highlight the syntenic *ftsH* gene pairs

To find the collinear relationship between members of the *NtftsH* gene family and *ftsH* genes of other species, we selected a dicotyledonous plant (Arabidopsis), a monocotyledonous plant (rice), and three Solanaceae plants (tomato, potato, and pepper). Collinear blocks of 5, 4, 15, 15, and 12 were identified in Arabidopsis, rice, tomato, potato, and pepper, respectively. Therefore, based on the collinearity between tobacco and these 5 plants, we have drawn 4 collinear diagrams representing homologous gene pairs of tobacco and other species, connected by blue lines (Fig. 5; Additional file 6 Table S4). There are five pairs of ftsH genes between tobacco and Arabidopsis, and 4 pairs of genes between tobacco and rice collinear. About 11 genes among the 20 identified *ftsH* genes showed no collinearity with either Arabidopsis or rice, suggesting that these homologous gene pairs may have formed after the divergence of dicots and monocots or after the divergence of the two species. In addition, a collinear pairing of the *ftsH* genes was found between tobacco and two other species, suggesting that this gene (*NtftsH*6) may have existed before evolutionary differentiation. We found that the *ftsH* genes in tobacco and three Solanaceae plants have high homology among the same family members; few genes without many homologies may have happened due to the independent expansion of the *ftsH* gene family in these plants.

### GO annotation analysis of *ftsH* gene

**Fig. 6:**
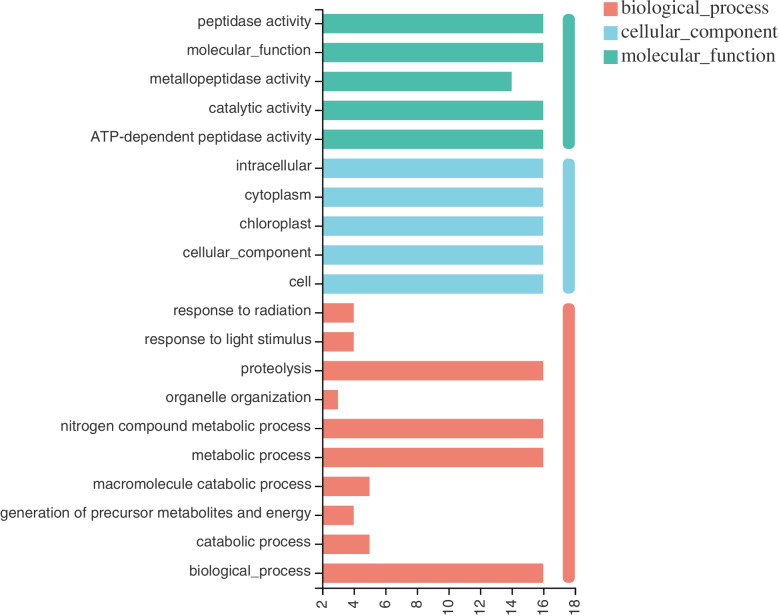
GO annotation analysis of 20 *ftsH* genes from tobacco; Green represents a molecular function, bright blue represents a cellular component, and orange represents a biological process

The Gene Ontology (GO) analysis was performed and the *AtftsH* protein sequence was used as a reference. The results showed that *NtftsH* protein might be involved in various biological, cellular, and molecular processes (Fig. 6; Additional file 7 Table S5). Analysis of the biological processes mediated by *NtftsH* protein showed that *NtftsH* protein is mainly involved in catabolic processes, precursor metabolites and energy production, macromolecular catabolic processes, nitrogen compound metabolism, organelle organization, protein breakdown, and responding to light stimuli, radiation, and cellularity. In addition, analysis of cellular components revealed that *NtftsH* protein is localized in intracellular organelles, including chloroplasts, mitochondria, and symplasts. The *NtftsH* protein also has various molecular functions, including ATP-dependent peptidase activity, catalytic enzyme activity, hydrolase activity, and metalloprotease activity.

### Gene tissue expression specific analysis

**Fig. 7:**
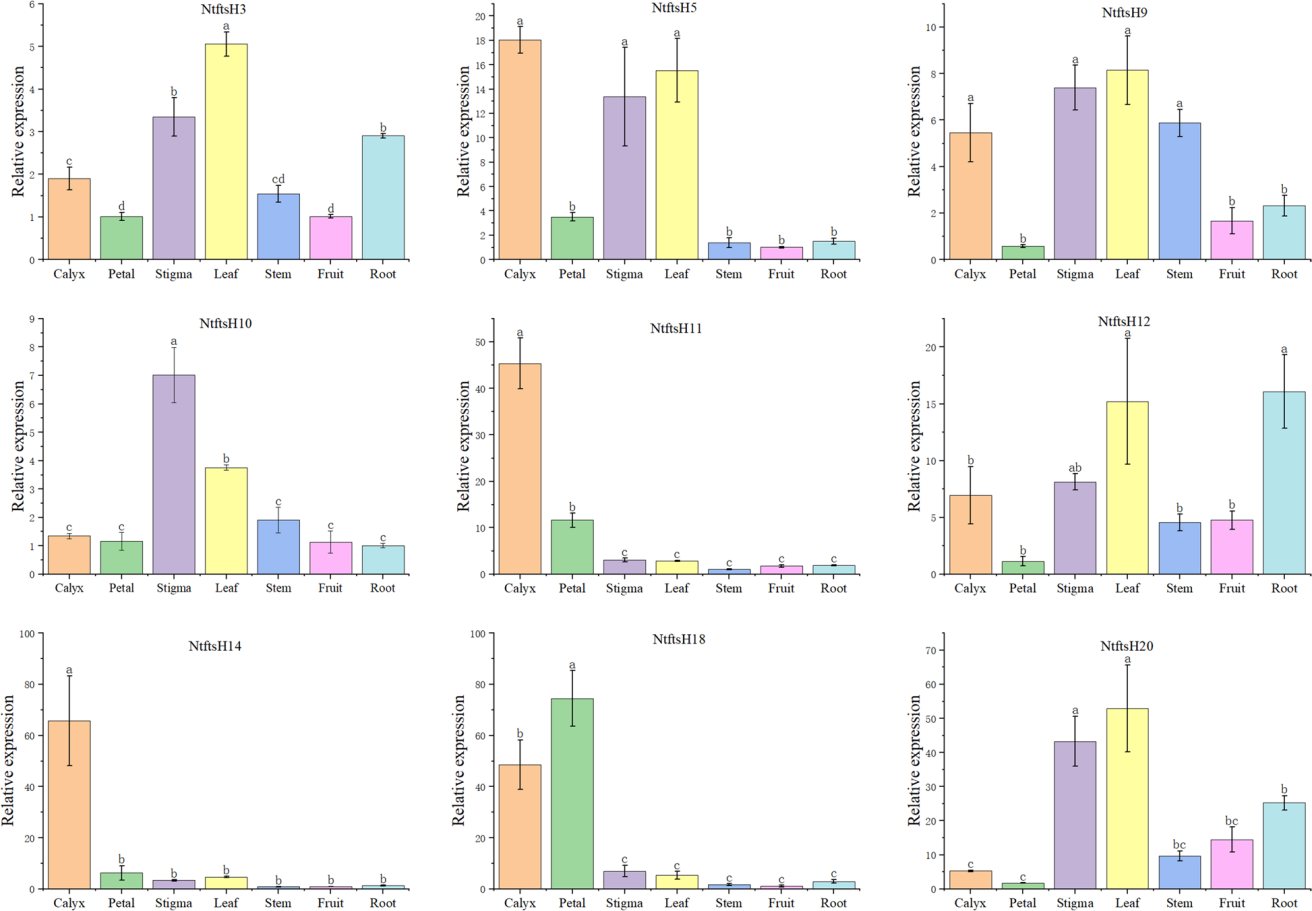
Expression patterns of nine *NtftsH* genes in different tissues of the tobacco plant; Expression patterns of 9 *Nicotiana tabacum* L. *ftsH* in the calyx, petal, anther, stigma, leaf, stem, fruit, and root organs, as examined by qRT-PCR. Error bars represent standard error. Lowercase letters above bars indicate a significant difference (*P* < 0.05, least significant difference test) among the treatments. *ftsH*: filamentous temperature-sensitive H; qRT-PCR: Real-time quantitative PCR

To study the physiological role of the *NtftsH* gene, we selected 9 out of 20 *NtftsH* genes to study their expression patterns. The expression levels of these 9 genes in 7 tissues (calyx, petals, stigma, leaves, stems, fruits, and roots) were detected by qRT-PCR (Fig. 7). The expression patterns of these genes in different organs of tobacco varied, indicating that *NtftsH* genes have diverse functions in tobacco. The selected 9 genes were expressed in all organs. The expression levels of *NtftsH*3, *NtftsH*9, *NtftsH*12, and *NtftsH*20 were higher in leaves than in other organs, and the amount of *NtftsH*5 in leaves was second only to calyx. In the calyx tissue, *NtftsH*5, *NtftsH*11, and *NtftsH*14 genes were highly expressed, and the expression levels of other genes were relatively low; *NtftsH*18 was highly expressed in the petal tissue. The expression of *NtftsH*10 was the highest in the stigma tissue, followed by *NtftsH*3, *NtftsH*5, *NtftsH*9, and *NtftsH*20. All nine genes were expressed in roots; *NtftsH*20 had the highest expression, followed by *NtftsH*12.

### Gene expression analysis under different stresses

**Fig. 8:**
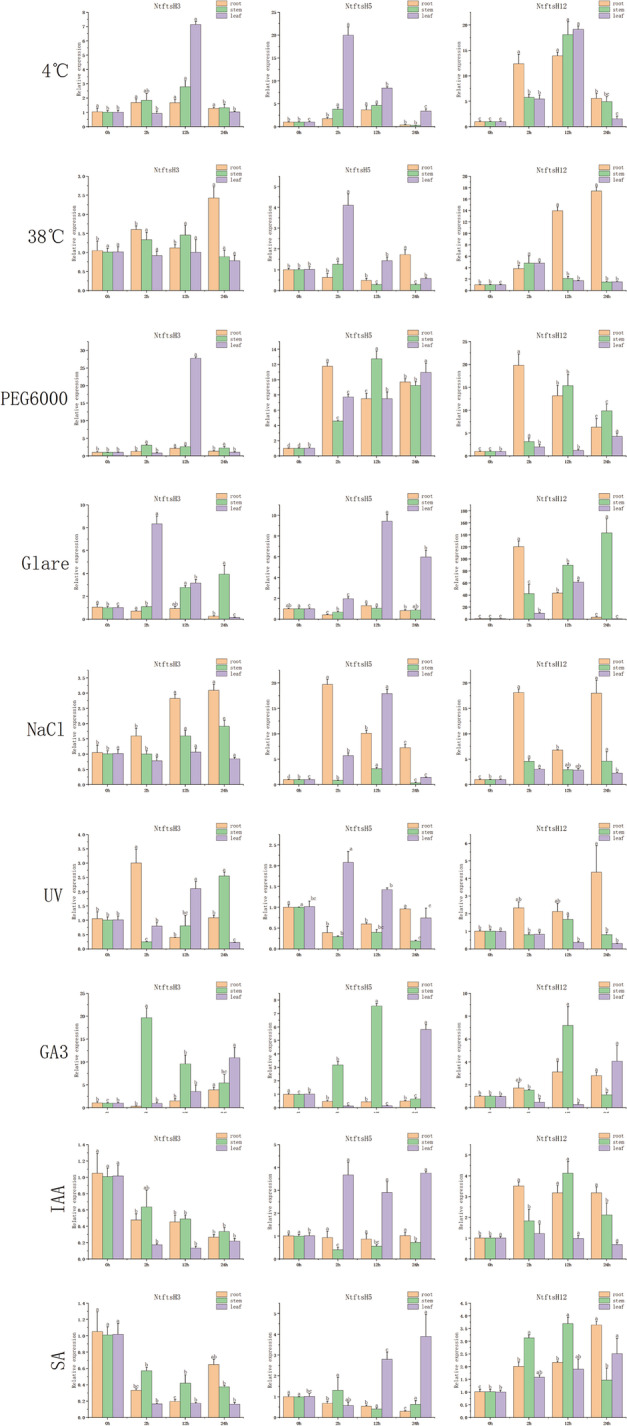
Expression of 3 *Nicotiana tabacum* L. *ftsH* in plants subjected to abiotic stresses (NaCl, strong light, PEG6000, UV, 38 °C, 4 °C, and hormones (100 μmol/L IAA, SA, GA3)) at the seedling stage. *ftsH*: filamentous temperature-sensitive H; qRT-PCR: Real-time quantitative PCR. Expression patterns of 3 *NtftsH* in root, stem, and leaf organs were examined by qRT-PCR. Error bars represent standard error. Lowercase letters above bars indicate a significant difference (*P* < 0.05, LSD) among the treatments

To study the expression of *NtftsH* under abiotic stress conditions, we used qRT-PCR to evaluate the expression of 3 *NtftsH* genes in roots, stems, and leaves. Around 9 abiotic stress situation was created with the following: polyethylene glycol (PEG), high-temperature, low temperature, strong light, high salt (NaCl), ultraviolet (UV) light, salicylic acid, auxin, and gibberellin treatments (Fig. 8). The expression patterns of each *NtftsH* gene were different under the 9 abiotic stress conditions; some genes were significantly up-regulated, and some genes were inhibited. The expression patterns of most genes changed significantly during the early stages of the stress response (Fig. 8); the expression of some *NtftsH* genes also changed over time or in different tissues under several stress treatments. For example, in response to 15% PEG6000, the expression of *NtftsH*3 in leaves reached a peak post 12 h of treatment and then gradually decreased; both *NtftsH*5 and *NtftsH*12 were up-regulated with prolonged treatment time, but the expression level of *NtftsH*12 was not as high as that of *NtftsH*5. The expression levels of all *NtftsH* in tissues showed a trend of initial up-regulation and then down-regulation; *NtftsH*5 and *NtftsH*12 also demonstrated this expression pattern in stem and leaf tissues. The low-temperature stress induced the expression of the three genes in roots, stems, and leaves, which also showed a trend of high production initially and reached a very significant level in leaves. Also, under the stress of high temperature, the expression of *NtftsH*12 was high in the stem at 24 h of treatment. Around 24 h after hormone treatment with IAA or SA, the expressions of *NtftsH*3 in various tissues were significantly down-regulated, *NtftsH*5 was up-regulated in leaves, and *NtftsH*12 was up-regulated in roots and stems.

## Discussion

*FtsH* gene is commonly present in all plants and plays an important role in plant growth and development, especially in the development of plant chloroplasts. This gene has been intensively studied in many plants, including tomato, potato, and rice plants. The genetic background of common tobacco is special, it is allotetraploid, and it is divided into ss and tt. The gene mutation in the evolution process and the gene recombination in the process of tobacco cross-breeding make the ss and tt blend, making the tobacco genetic background more complex, so the analysis of the tobacco *FtsH* gene family is very important [30]. The *ftsH* gene family has been widely studied in many species based on genome-wide analysis. In this study, 20 genes were identified as members of the *ftsH* gene family in the tobacco genome; they were named according to their distribution and location on the chromosome, from *NtftsH1* to *NtftsH20*. Similar numbers of *ftsH* genes were found in other plants even though genome size varies between species. For example, rice has 9 and Arabidopsis has 12 *ftsH* genes. This indicates that the number of *ftsH* family members is relatively stable and has no absolute correlation with genome size.

In the process of plant evolution, the appearance and expansion of introns are related to gene family expansion and subsequent acquisition of new gene functions; introns usually appear in the early stage of gene family expansion but gradually disappear over time [31–33]. We found that introns are widespread in the *ftsH* gene through the study of gene structure. The number of introns in the 20 *NtftsH* genes ranged from 3 to 27. The structure of the *NtftsH* gene is similar to that of Arabidopsis, pear, and other plants, and this indicates that the *FtsH* gene is highly conserved among different species. Some of these genes contain large-span introns, which may be recently evolved *NtftsH* genes. *NtftsH* genes clustered on the same phylogenetic branch exhibited similar intron–exon composition and conserved domains, suggesting that they perform similar functions in plants. Notably, *NtftsH* shows similarity in structure and conserved domains with members of the *ftsH* gene reported in rice, pear, and apple [23]. At the same time, some *NtftsH* gene members contain the FtsH_ext domain at the N-terminus, and this change in the structure could be due to the altered function of the *NtftsH* gene. In addition, the conservation of the ftsH domain maintains the basic functions of the gene family, as diversity reduces the selection pressure during evolution [34].

Gene duplication plays an important role in biological evolution and the expansion of gene families. This study found that the *NtftsH* gene had 4 pairs of segmental duplications. Similar to a previous study on the pear tree [23], no tandem duplication of the *NtftsH* gene was found in the tobacco genome. These results suggest that segment duplication may contribute more to the expansion of the *NtftsH* family than tandem duplication. This is consistent with previous studies on other plant species, and segment duplication events may have contributed to the formation and evolution of some *NtftsH* genes. Out of 24, only 13 chromosomes harbor *NtftsH* genes, and there was no correlation between the distribution of the *NtftsH* gene and the density of genes on the chromosomes. This suggests that the *NtftsH* gene family was affected by gene deletion during the evolutionary process. Therefore, some *ftsH* genes may have arisen by duplication events, suggesting that duplication events played an important role in the rapid expansion of *ftsH* family members in plants.

Due to the complexity of biological research and the increasing scale of biological research, Gene Ontology provides a comprehensive description method for gene function and its products in order to describe the gene products uniformly. We performed GO analysis on the *NtftsH* gene based on the Arabidopsis protein sequence. *NtfsH* is structurally similar to rice, Arabidopsis and other plants, which may lead to their similarities in molecular functions. *FtsH* proteins are ATP-dependent active enzymes and possess hydrolase activities as well. According to the GO annotation results, the *ftsH* gene is a component of important cellular organelles such as chloroplasts and mitochondria, and is also involved in the catabolism of PSII-related light-harvesting complexes, which is similar to the studies of Arabidopsis, rice, bayberry and other plants [16, 35–37]. Deg1 protein in photosynthesis photosystem II was degraded to prevent damage to leaves under strong light conditions, and knockout of the rice *ftsH* gene resulted in an albino phenotype of the F2 generation, indicating that the *ftsH* gene is involved in the synthesis of chloroplasts [7, 8, 11]. The GO annotation results showed that the *ftsH* is also involved in the development of plant embryos and leaves similar to the function of *AtftsH* reported in Arabidopsis plants [38, 39], further confirming the accuracy of the GO annotation results. Many elements related to chloroplast and photosynthesis are enriched in the annotation of *NtftsH* molecular functions, indicating that the ftsH gene is closely related to photosynthesis functions. And it has been confirmed in cyanobacteria, Arabidopsis, bayberry, rice, corn and other organisms that perform photosynthesis [7, 9–11, 24, 40].

Analysis of gene expression profiles can provide a better understanding of the potential biological functions of genes. To understand the role of *NtftsH*, we selected 9 *NtftsH* genes with significant structural differences to study their expression patterns in different tissues. These 9 *NtftsH* genes were expressed in all organs tested, with relatively high expression levels in calyx, leaves, and roots indicating its role in growth and development — this result corroborates with the findings in Arabidopsis and rice.

Several studies have shown that the *NtftsH* gene plays an important role in plant stress response [41–44]. We also investigated the gene expression patterns of three *NtftsH* genes under nine abiotic stress conditions. The results showed that the expression of most *NtftsH* genes varies under different abiotic stress situations. For example, under drought stress, the expression of *NtftsH*5 was up-regulated in roots, stems, and leaves; the expression of *NtftsH*12 was up-regulated first and then down-regulated in roots, stems, and leaves. Interestingly, the expression of the same genes showed an opposite profile under other stress conditions. In stems, the expression of the *NtftsH*3 gene was up-regulated under high-temperature stress but remained unchanged under cold stress. These results suggest that *NtftsH* genes are involved in the plant’s response to abiotic stress and participate in a complex cross-regulatory network in plants. When the plants were subjected to cumulative abiotic stresses such as PEG, high temperature, low temperature, strong light, and NaCl, the expression levels of these three genes changed significantly in different tissues. This indicated that they may be co-expressed under various stress conditions. This is confirmed with the results of the cis-acting element analysis, which shows that the NtftsH gene contains a large number of cis-acting elements related to light and temperature stress. It may be that these cis-acting elements and their corresponding transcription factors are activated under stress, thereby regulating the expression of the NtftsH gene. Previous studies have shown that the *ftsH* gene is an important regulatory protein in plant response to high light stress. Therefore, the *NtftsH* gene may have functions similar to the *ftsH* genes of other plants [7, 8, 44]. Follow-up studies can verify the function of the *NtftsH* gene and assess its ability to resist stress conditions in different varieties of tobacco plants.

In conclusion, this study provides a comprehensive survey of the *NtftsH* genes as a reference to studying the ftsH genes' function. In this study, we verified the potential role of the *NtftsH* gene in the process of the plant facing adversity in the form of various stress by analyzing gene expression profiles. However, the role of the *NtftsH* gene in response to stress and other biological processes requires further studies.

## Conclusion

A comprehensive analysis of the tobacco ftsH gene family was performed in this study. The identified 20 ftsH genes were renamed and divided into different groups according to the classification of AtftsH in Arabidopsis and OsftsH in rice and the evolutionary relationship between different members. The exon–intron structure and motif composition of the *NtftsH* genes in the same evolutionary branch show high similarity. Homology analysis and phylogenetic comparison with *ftsH* genes from several other plants provided valuable clues for studying the evolutionary characteristics of *NtftsH* genes. The *NtftsH* gene plays an important role in plant growth and development, as indicated by its expression patterns in different tissues and under different treatment conditions. Phylogenetic and gene expression analyses will provide the basis for the functional analysis of the *NtftsH* gene in the future. The results of this study offer a valuable resource for a better understanding of the biological role of the *ftsH* gene in tobacco.

## Methods

### *FtsH* gene identification

We downloaded the Hidden Markov Model (HMM) file corresponding to the *FtsH* domain (PF06480) from the Pfam protein family database (http://pfam.xfam.org). The *ftsH* gene was searched from the tobacco genome database using HMMER 3.0 (http://hmmer.org/), with default parameters, and the cut of was set to 0.01[45]. All candidate genes that may contain the *ftsH* domain were shortlisted based on HMMER 3.0 analysis; Pfam and SMART programs were then used to confirm the *ftsH* core sequence [46–48]. Each potential gene was then manually inspected to ensure that conserved sequences in the N-terminal region of the *ftsH* domain conformed to the predicted sequences. The wrongly predicted genes were manually screened out, some genes were verified by PCR amplification and sequencing, and redundant sequences were manually discarded. After the comprehensive screening, 20 *NtftsH* genes were finally identified in the tobacco genome, and then they were renamed according to the order and position of the genes on the chromosome [49]. The physicochemical properties such as molecular weight (MW) and isoelectric point (pl) of *NtftsH* family members were predicted and analyzed using the online analysis software ExPASy (https://web.expasy.org/)[50]. Subcellular localization prediction was performed using Cell-Ploc2.0 (http://www.csbio.sjtu.edu.cn/bioinf/Cell-PLoc-2/)[51].

### Sequence and phylogenetic analyses of *ftsH* genes

We used the ClustalW default parameters to align the protein sequences of *ftsH* from tobacco and *Arabidopsis thaliana*. After the null members were removed, the phylogenetic trees were constructed in MEGA 11.0, based on the equally weighted neighbor-joining method. Bootstrap values of the phylogenetic tree were calculated with 1000 replicate analyses. The amino acid sequence of the deduced *ftsH* domain was then manually adjusted using GeneDoc software. We used the online website Gene Structure Display Server (GSDS: http://gsds.cbi.pku.edu.cn) to compare the predicted coding sequence with the corresponding full-length sequence to determine the exon–intron of the *NtftsH* gene structure [52]. Conserved domain analysis was performed by MEME (Multiple Expectation maximizations for Motif Elicitation;http://meme-suite.org/tools/meme) online software, the number of motifs was set to 10, and the range of motif width was set to 50–100 amino acids [53].

### Chromosomal distribution and analysis of promoter *cis*-acting elements

The tobacco annotation information (https://solgenomics.net/), present in the Solanaceae database, helped to identify the beginning of the *ftsH* gene on the chromosome. The duplication events of the genes were assessed by comparing the two genes. The *NtftsH* genes were mapped to chromosomes using the online tool MapGene2Chrom web v2 (http://mg2c.iask.in/mg2c_v2.0/) after determining their chromosomal positions from the tobacco genomic database. The 2000 bp upstream gene sequence of *NtftsH* was intercepted from the Solanaceae database, and the *cis*-acting elements were analyzed by screening the PlantCARE database (to identify plant *cis*-acting regulatory elements).

### Gene duplication and gene collinearity analysis

Gene duplication events were analyzed using the multicollinear scanning tool (MCScanX) [54]. The *ftsH* collinear analysis map was constructed by Dual Systeny Plotter software, and the *ftsH* collinear relationship between tobacco and pepper, tomato, potato, rice, and Arabidopsis were determined separately.

### Plant material and its handling

*Nicotiana tabacum* L. (K326) seeds, provided by the Key Laboratory for Tobacco Quality Research Guizhou Province, were used as the experimental material. Tobacco seeds were sown on a special substrate for flue-cured tobacco with the following conditions: 75% relative humidity, temperature 28 °C during the day (16 h) and 20 °C at night (8 h), and floating seedlings. About 80 days after transplanting, the calyx, petals, anthers, stigmas, leaves, stems, fruits, and roots of 3 similar healthy plants were collected; this sampling was repeated three times. We selected 3 *NtftsH* genes to study the expression patterns of *NtftsH* genes under different abiotic stress conditions. With the emergence of the sixth leaf, the plants showing similar growth vigor were selected and subjected to stress conditions as follows: salt (5% NaCl), strong light (1000 μmol m^−2^ s^−1^), drought (15% PEG6000), UV, high temperature (38 °C), low temperature (4 °C), and hormones (100 μmol/L IAA, SA, GA3). Three replicates were used for each abiotic stress treatment; samples were collected after 0 h, 2 h, 12 h, and 24 h of treatment. The samples were either immediately frozen in liquid nitrogen or stored in an ultra-low temperature freezer at –80 °C for subsequent qRT-PCR analysis.

### Extraction of the total RNA from the plant material, reverse transcription of cDNA, and analysis by qRT-PCR

Total RNA was extracted from the sample with RNAprep Pure Plant Kit (TIANGEN, DP432), and its quality and concentration were detected by agarose gel electrophoresis and concentration detector, respectively. cDNA was obtained using FastKing gDNA Dispelling RT SuperMix kit (TIANGEN, KR118) following the manufacturer's instructions. We selected nine *NtftsH* genes to study gene expression patterns. qRT-PCR primers were designed by Primer Premier, version 6 (Additional file 5: Table S5) to analyze gene expression. We used the Actin gene as an internal PCR control because it is stably expressed in almost all tissues at every growth phase. We performed the qRT-PCR analysis with Talent qPCR PreMix (SYBR Green; TIANGEN, FP209). Each reaction was performed in three biological replicates, and the final detection result was calculated by the 2^–ΔΔCt^ method [55].

### Statistical analysis

SPSS (Statistical Product and Service Solutions) software was used to analyze the variance of the data, and data were compared with the least significant difference test at the 0.05 and 0.01 levels. OriginPro 2021 software was used for the generation of graphs.

## Supplementary Information

**Additional file 1:** **Table S1.** List of the 20 NtftsH genes identified in this study.

**Additional file 2:** **Figure S1.** Multiple sequence alignment.

**Additional file 3:** **Figure S2.** Chromosomal location of NtftsH gene. The distribution of the NtftsH gene on chromosomes. Yellow indicates the ID of the chromosome. Taking 300 kb as the genetic interval, the color gradient from red to blue on the tobacco chromosome corresponds from high density to low density. Chromosomal blank regions represent genetic regions lacking gene distribution information.

**Additional file 4:** **Table S2.** List of the tandem repeat gene pairs of ftsH in tobacco.

**Additional file 5:** **Table S3.** Summary of cis-elements is in the promoter regions of ftsH family genes in tobacco.

**Additional file 6:** **Table S4.** The lists of tobacco and other species ftsH synteny gene pairs.

**Additional file 7:** **Table S5.** GO annotation of NtftsH.

**Additional file 8:** **Table S6.** The primer sequences for qRT-PCR used in this study.

**Additional file 9:** **Table S7.** Gene expression data and cq value under different stress treatments.

## Data Availability

The whole *Nicotiana tabacum* L. genome sequence information was obtained from the Sol Genomics website (https://solgenomics.net/organism/Nicotiana_tabacum/genome) and the website is open to all researchers. *Nicotiana tabacum* L. (K326) seeds, provided by the Key Laboratory for Tobacco Quality Research Guizhou Province, were used as the experimental material. The datasets supporting the conclusions of this article are included in the article and its Additional files.

## Abbreviations

*ftsH*: Filamentous temperature-sensitive H
CDS: Coding sequence length
SPSS: Statistical Product and Service Solutions
MEGA: Molecular Evolutionary Genetics Analysis
MEME: Multiple Expectation maximizations for Motif Elicitation
Mw: Protein molecular weight
PEG: Polyethylene glycol
pI: Isoelectric point
qRT-PCR: Real-time quantitative PCR
SMART: Simple Modular Architecture Research Tool
UV: Ultraviolet
