# Supplementary figures and images for: Genome-wide identification and expression analysis of the ftsH protein family and its response to abiotic stress in Nicotiana tabacum L
Source: BMC Genomics. 2022 Jul 12;23:503. doi: 10.1186/s12864-022-08719-x (PMC9281163; doi:10.1186/s12864-022-08719-x)

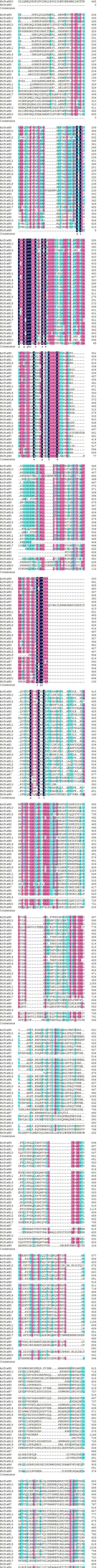

Supplement: Supplementary file 2 — Additional file 2: Figure S1. Multiple sequence alignment. [file 12864_2022_8719_MOESM2_ESM.png]

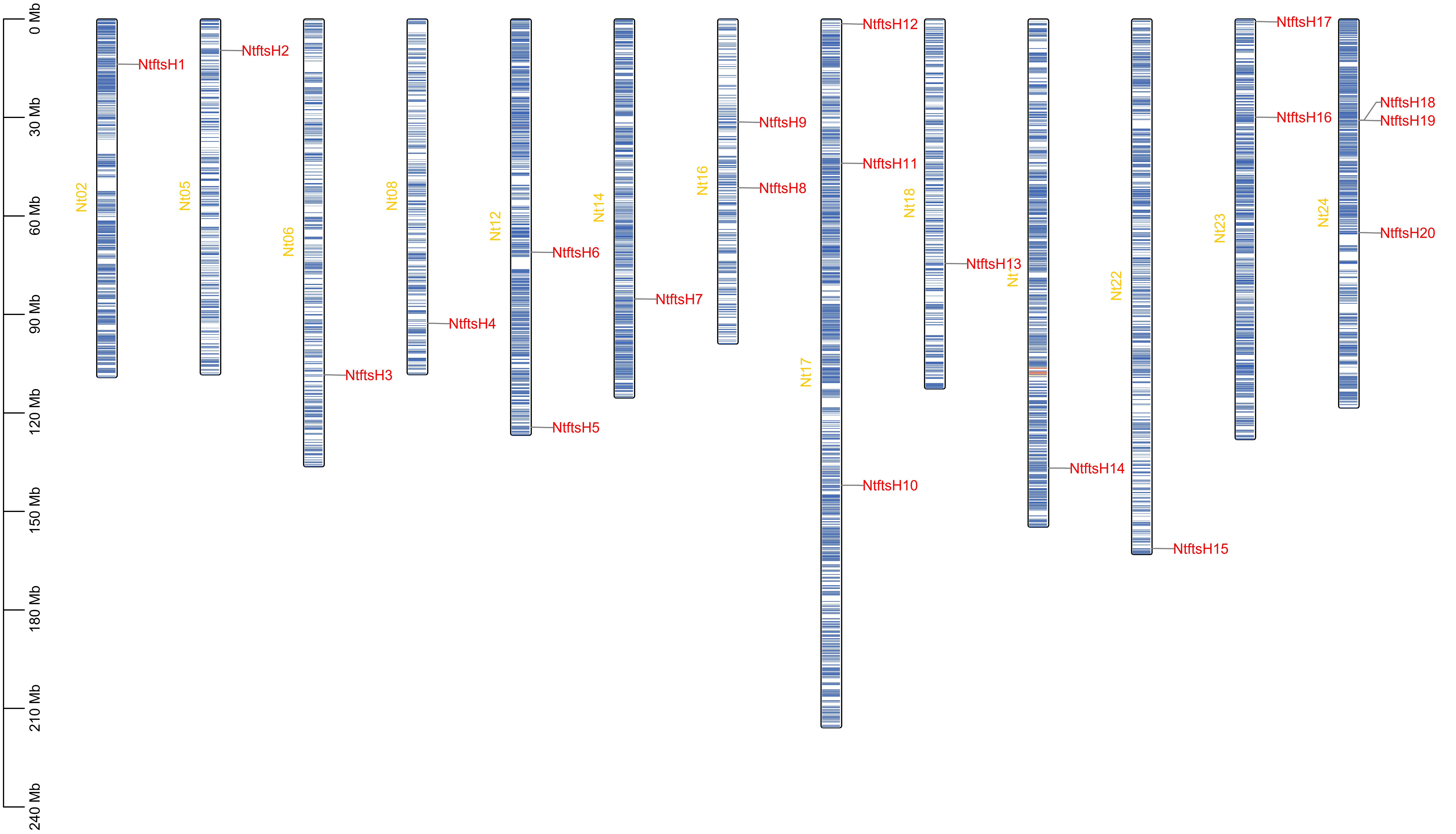

Supplement: Supplementary file 3 — Additional file 3: Figure S2. Chromosomal location of NtftsH gene. The distribution of the NtftsH gene on chromosomes. Yellow indicates the ID of the chromosome. Taking 300 kb as the genetic interval, the color gradient from red to blue on the tobacco chromosome corresponds from high density to low density. Chromosomal blank regions represent genetic regions lacking gene distribution information. [file 12864_2022_8719_MOESM3_ESM.jpg]
